# Breast Hamartoma: Mammographic Findings

**DOI:** 10.5812/iranjradiol.4492

**Published:** 2011-12-25

**Authors:** Donya Farrokh, Janbakhsh Hashemi, Emad Ansaripour

**Affiliations:** 1 Department of Radiology, Imam Reza Hospital, Mashhad University of Medical Sciences, Mashhad, Iran

**Keywords:** Giant Mammary Hamartoma, Mammography, Breast

Dear Editor,

Breast hamartoma is an uncommon, benign, slow-growing lesion found in all age groups after puberty. This pathological entity has a reported incidence of 1.2% of benign lesions and 4-8% of benign breast tumors in women [1][2]. It appears that with the increasing use of breast screening more hamartomas are likely to be identified.

This rare pathological entity consists of a well-circumscribed, benign tumor, composed of variable amounts of fat, fibrous and glandular tissue that may contain various benign changes such as fibrocystic changes. Breast hamartomas usually present as painless mobile breast lumps in middle-aged women. Up to 60% of these benign breast lesions are soft and non-palpable [2].

The various amount of fat and fibrous tissue within the breast hamartomas results in different mammographic appearances. The characteristic mammographic features of hamartoma are that of a nonhomogeneous compressible mass with dense nodules of fibrous connective tissue interchanged by a thin radioopaque pseudocapsule. The pseudocapsule results from displacement of breast parenchyma by the tumor [3][4]. Hamartomas that contain predominantly fibrous tissue appear uniformly dense. In these patients, hamartomas can not be differentiated from fibroadenomas that generally have glandular homogeneous density within a thin halo of compressed fat. Hamartomas containing predominantly fatty tissue may mimic lipoma, fat necrosis and oil cyst [4][5].

Lobulated densities that may be dispersed within the encapsulated fat, described as a “slice of salami” [3][5]. Calcification is a rare mammographic finding, but amorphous or smooth and round microcalcifications are reported. It is important to keep in mind that pleomorphic calcification, spiculated opacity or a well-demarcated dense opacity in a hamartoma are suspicious for malignancy [6][7].

**Figure 1:**
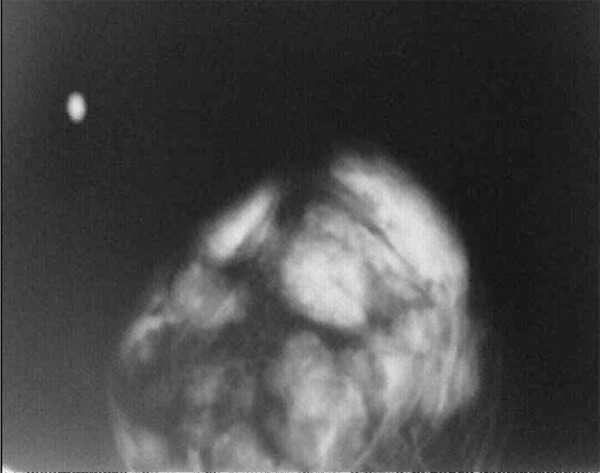
Mammography shows the appearance of a slice of salami.

**Figure 2:**
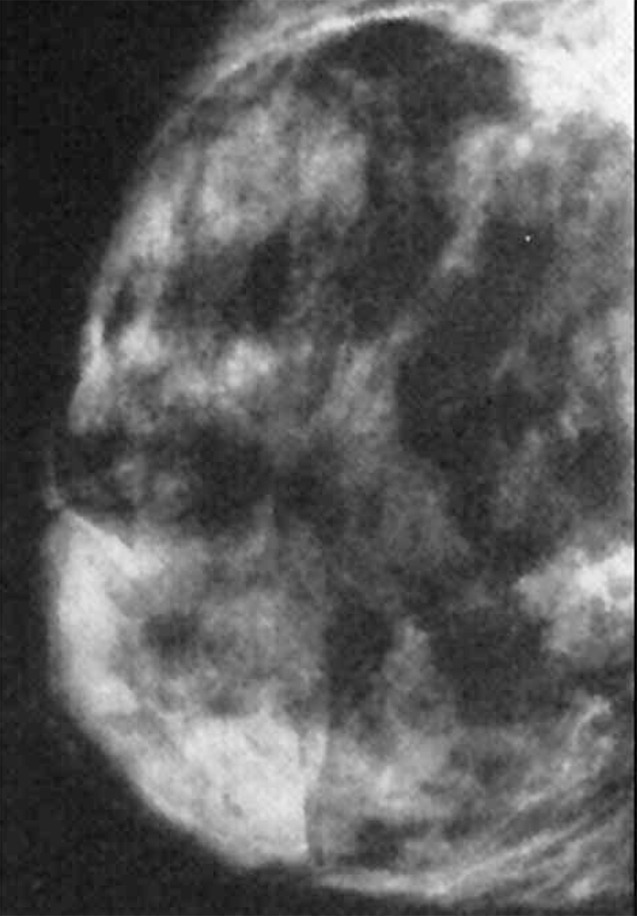
Mammography shows a large non-homogeneous mass.

**Figure 3:**
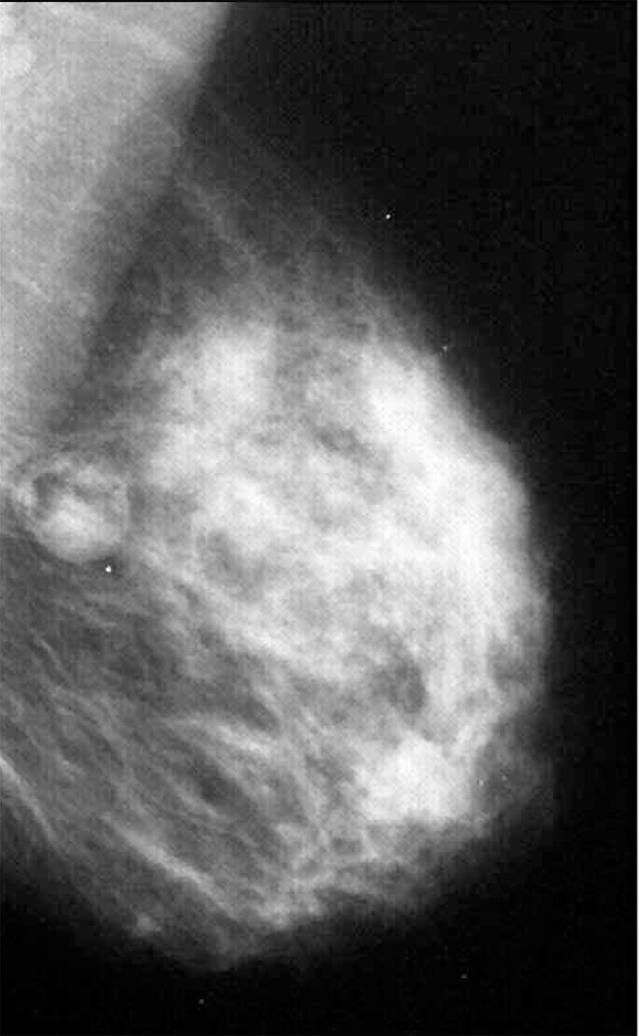
Mammography shows a dense mass containing fatty tissue.

**Figure 4:**
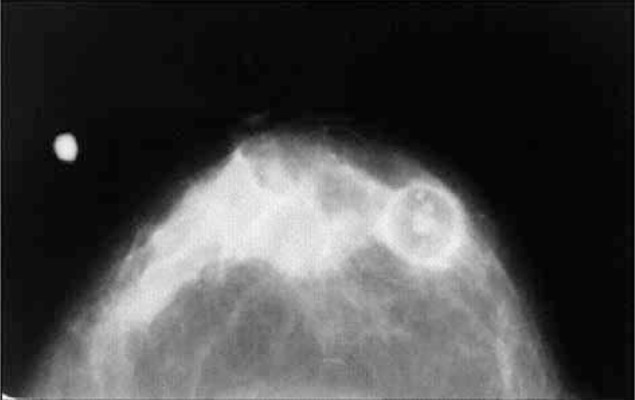
Mammography shows a uniformly dense mass.

Twenty-five patients with breast hamartomas were diagnosed based on mammography and pathologic findings during a ten-year period from July 1998 to July 2008. The patients’ age ranged from 28 to 60 years. Nine of the cases had non-palpable lesions as a result of their small size or consistency that was similar to that of the surrounding breast tissue. These cases were selected from the group of patients with lesions detected in the screening program. Clinically, 16 lesions were palpable masses which were well-defined and of soft consistency. All patients underwent preoperative mammography. The most common mammographic findings of the breast lesions were an oval-shaped (12/25), smooth and well-defined mass (20/25), internal fat densities (13/25) and radiolucent halos (10/25). In only 13 patients (52%) the classic mammographic appearance of breast hamartoma was seen (Figure 1 and 2). Nine of 25 patients (36%) presented a mass of mixed radiographic density of fat and soft tissue with soft tissue predominance (Figure 3) and a dense mass was seen in the remaining cases (Figure 4). We detected a benign appearing calcification mammographically in one patient, but calcification was demonstrated on histological examination in four patients.

The variable amount of fat and fibrous tissue within a hamartoma may result in a wide spectrum of sonographic appearances and due to their variability the role of ultrasound in the diagnosis of breast hamartoma has been minimal. The most common sonographic appearance is a well-circumscribed solid hypoechoic mass with non-homogeneous echogenicity and irregular hyperechoic nodules or bands between these hypoechoic areas. Hamartoma may contain echolucent areas due to homogeneous adenomatous portions surrounded by echogenicity from interfaces of fibrous and adipose tissue between the adenomatous portions [8][9]. When a mammographic round or oval mass with a heterogeneous internal density and a radiolucent halo becomes a compressible heterogeneous mass which is surrounded by an echogenic or echolucent halo on sonography, breast hamartoma may be diagnosed.

Because of the lack of cytological and architectural specificity for breast hamartoma and diagnostic difficulties in the cases in which both mammographic and ultrasonographic findings are atypical, the correlation between clinical manifestation, imaging findings and histology is essential for the successful diagnosis of breast hamartoma.

Hamartomas do not possess specific diagnostic histological features; therefore, pathological diagnosis is usually difficult. The presence of fibrous tissue within the lobules, or fibrous tissue and fat in the stroma with or without angiomatous changes should alert the pathologist to the possibility of hamartoma. The presence of breast lobules and ducts help to distinguish this tumor from fibroadenoma in which lobules are often absent [2][4].

As with fibroadenoma, which has a reported incidence of 0.1% for malignant changes, it is likely that carcinoma arising in a breast hamartoma is a coincidental finding. Malignancy may arise in the glandular tissue of hamartoma [7].

We did not perform histological examination in the nine cases of breast hamartoma with typical mammographic appearance. Histological examination was carried out on the other 15 cases because of the atypical appearance of a large mass in mammography, breast deformity, positive familial history for breast cancer and cancer phobia. There is no evidence of malignancy in patients with pathologically proven breast hamartoma.

A lesion with characteristic morphologic appearance of hamartoma should not be recalled and excision biopsy is unnecessary in these patients. Surgical intervention should be reserved for cases found to be inconclusive after imaging modalities and core biopsy examination for patients with a large mass and breast deformity or women with cancer phobia and also for those patients who have clinical signs of a malignant lesion.

The current trend of mammographic breast screening has made us aware that indeed breast hamartomas are not uncommon lesions. The mammographic appearance of breast hamartoma is variable and the classic mammographic appearance of this lesion is less common than previously reported. These tumors may go unrecognized by the pathologists if the clinical impression of a distinct breast lump or breast asymmetry and the imaging features are not taken into consideration, because pathology shows all the consistencies of the normal breast tissue.
